# Tracking the dynamic changes of a flavor, phenolic profile, and antioxidant properties of *Lactiplantibacillus plantarum*‐ and *Saccharomyces cerevisiae*‐fermented mulberry wine

**DOI:** 10.1002/fsn3.2590

**Published:** 2021-09-22

**Authors:** Jie Hu, Annadurai Vinothkanna, Meng Wu, John‐Nelson Ekumah, Nelson Dzidzorgbe Kwaku Akpabli‐Tsigbe, Yongkun Ma

**Affiliations:** ^1^ School of Food and Biological Engineering Jiangsu University Zhenjiang China

**Keywords:** antioxidant, fermentation, flavor characters, mulberry wine, phenolics

## Abstract

The process of fermentation renders the superior quality of mulberry wine based on the microorganisms utilized. The present study aimed at investigating the changes and correlation between phenols and product quality of mulberry wine fermented with *Lactiplantibacillus plantarum* and *Saccharomyces cerevisiae* combinatorially. Total anthocyanins concentration (TAC), polyphenols concentration (TPC), flavonoids concentration (TFC), and antioxidant capacity decreased significantly with high correlation in the fermentation process. TAC gradually reduced with a loss rate of 47.98% from 0 to third day of fermentation. Fermented mulberry wine obtained indicated a dynamic balance due to the presence of *p*‐hydroxybenzoic acids as compared to the primary phenolic constituent. Chlorogenic acid usually presents in mulberry juice. The relative intensity of sourness was the most prominent and reached the maximum (10.93) on day 2 of fermentation. A total of 21 volatile esters were quantified (6621.59 μg/L), which contributed significantly to the aroma of mulberry wine. The enhanced quality of fermented mulberry wine showed contraindications with decreased constituents and escalated wine quality. Rather than usual single inoculum, fermentation combination of LAB and yeast holistically influenced the color, taste, fragrance, phenolic profiles, and antioxidant properties in mulberry wine, ensuring palatability and fit for commercialization prospects.

## Graphical abstract

Active substances and the antioxidant capacity of mulberry wine decreased in the fermentation process. Esters and alcohols were the main volatile substances in the aroma of mulberry wine. The taste turned to sour at the end of fermentation. Combination of LAB and yeast fermentation holistically influenced the color, taste, fragrance, phenolic profiles, and antioxidant properties in mulberry wine, ensuring palatability and fit for commercialization prospects.

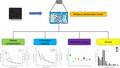

## 1 INTRODUCTION

Mulberry fruit belonging to the Moraceae family is ubiquitously present and localized widely across the Europe, Africa, and south‐east Asian countries (China, Japan, Korea, India and the Himalaya foothills), and more prevalent in China (Bao et al., 2016). Traditional Chinese Medicine has witnessed the initialization of therapeutic potentials of food as medicine and has been documented in the Chinese pharmacopeia edition 2015 (Zhang, Ma, et al., 2018). Mulberry poses a rich and nutritious bioactive ingredients including vitamins, minerals, fibers, amino acids, polysaccharides, and a variety of polyphenols, flavonols and phenolic acids, as well as anthocyanins (You et al., 2018; Yuan & Zhao, 2017). A wide spectrum of bio‐pharmacological activities has been emphasized, comprising antioxidant activities, anticancer profiles, cardiovascular abatement, immunomodulatory effects, hepatoprotective efficacies, antihyperglycemic perspectives, and neuroprotective properties (Khalifa et al., 2018; Sanchez‐Salcedo et al., 2015). Further, the polyphenols in mulberry have been addressed as beneficiary in combating diabetes, weight loss, and anti‐inflammatory potentialities due to wine compositions (Li et al., 2017; Mahboubi, 2019; Vasserot et al., 2010; Wei et al., 2018). The colorants in phenolic dark brown fruits belonging to natural polyphenols play a decisive role, and cyanidin 3‐glucoside (C3G) and cyanidin 3‐rutinoside (C3R) are the major constituents in mulberry fruit compositions. (Juan et al., 2012). Phenolic acids in mulberry are attributed to hydroxycinnamic acid and benzoic acid derivatives (Yuan & Zhao, 2017). The mulberry plant and various functionalities pertain with essential functions of fruit, leaves, and the root bark establishing versatility (Kavitha & Geetha, 2018; Zhang, Ren, et al., 2014). Maintaining the perseverance of mulberry fruits with concomitant functional reservations renders the post‐harvest elongations optimally (Wang, Sun, et al., 2015). Commercialization aspects of consumer perspectives are time‐consuming, and post‐harvest losses are the dire need. Hence, a rational approach in mulberry wine making is addressed as an important provocation. To further, an important provision is to optimize and provide a flavoring and aromatic optimistic potential in wine breweries.

Malolactic fermentation is affirmative of the present perspectives, and a tackling aspect is presented here to evaluate to the alcohol fermentation to mitigate the post‐harvest losses. The native preservatives in mulberry wine are incumbents of native aroma of wine brewing industrial scenario (Tchabo, Ma, Kwaw, Zhang, Xiao, et al., 2017). Resultant metabolites of monolactic fermentation yield a wide range of alcoholic secondary metabolites including alcohols, esters, acids, aldehydes, and carbonyl compounds which are produced in the process of alcoholic fermentation comprising a majority of prominence (Feng et al., 2015). Esterification and alcohol accumulation are an intrinsic preparations mechanism in fermentation, owing to the finalization. Economic feasibility is redressed in rendering the applicability and suitability of fermentative microbes with escalated efficiency. With a notion for aroma and flavor, wine industry necessitates the optimization protocols based on preliminary studies.

Winery researches are established based on the color, antioxidant capacity, and volatility of organic ingredients (Kwaw, Ma, Tchabo, Apaliya, Sackey, et al., 2018; Liu et al., 2017; Tchabo, Ma, Kwaw, Zhang, Xiao, et al., 2017). Research concerned with variation profiles of the change of phenolic constituents and holistic quality of the whole mulberry wine fermentation process are scarcely available. However, the active ingredients invigorated the changes in several active compounds during the mulberry alcohol fermentation pose process deserve attention dire benefits due to because they are vital for primary implications in human health. Earlier reports concerning the changes in bioactive phenolic compounds investigated during mulberry wine fermentation are adequately examined, and changes in flavor, quality, and texture remain to be the area unexplored (Wang, Sun, et al., 2015). Therefore, the present study aimed to discover the dynamic changes of total anthocyanin concentration (TAC), total polyphenol concentration (TPC), total flavonoids concentration (TFC), phenolic acid, and sensory parameters (flavor, color, and taste) during the process of mulberry wine fermentation. The correlation between phenolic substances, antioxidant capacity, and color. The preliminary analysis emphasizes the theoretical basis for further optimization and essential downstream processing.

## 2 MATERIALS AND METHODS

### 2.1 Plant material

Zhèn shēn 1 hào (*Morus nigra*) was picked from Jiangxinzhou, Zhenjiang, Jiangsu Province, China. The matured black fruits were harvested and selected for wine preparation. The surface microbial contamination was eliminated by treating sodium hypochlorite (0.02%, v/v) and sterile water. Subsequently, undamaged fruits were stored in the deep freezer at −20°C.

### 2.2 Chemicals

Phenolic acid standards (gallic acid, *p*‐hydroxybenzoic acid, protocatechuic acid, vanillic acid, syringic acid, chlorogenic acid, caffeic acid, *p*‐coumaric acid, ferulic acid, and erucic acid) were purchased from Shanghai Yuanye Biotechnology (Shanghai, China). Other analytical pure reagents were obtained from Sinopharm Chemical Reagent (Shanghai, China).

### 2.3 Mulberry wine fermentation process

#### 2.3.1 Activation of microbial species

The *Lactiplantibacillus plantarum* and *Saccharomyces cerevisiae* were procured from DuPont (Shanghai, China). Starter culture activation was preceded as per the method enumerated, previously Kwaw, Ma, Tchabo, Aapaliya, Wu, et al. (2018). Briefly, L. plantarum (37°C) and S. cerevisiae (35–40°C) were activated in 5% glucose solution for 20–30 min.

#### 2.3.2 Fermentation process

The stored frozen mulberry fruits were defrosted at room temperature (28 ± 2°C). After crushing and treatment with 0.1% pectinase, they were pretreated for temperature regulation by incubating in water bath at 40°C for 40 min duration and centrifuged (3200 g, 4°C for 10 min). The clarified juice was added with 60 mg/L SO_2_ and the fermentation was initiated by inoculating yeast (5%) and LAB (0.3%) at 22°C. Periodic samples of the fermentation broth were taken for further investigation on days 0, 1, 2, 3, 4, 5, 6, 7, 9, 11, 14, and 17.

### 2.4 Color measurement

Samples’ color parameters were measured with a colorimeter (HunterLab, Reston, USA) and expressed as the L*, a*, and b*. Based on a* and b*, the saturation C* and hue Angle *H* ^0^ of the samples were respectively calculated according to Equations (1) and (2) (Kwaw, Ma, Tchabo, Aapaliya, Wu, et al., 2018).

(1) $$C^{*}={\left[{\left(a^{*}\right)}^{2}+{\left(b^{*}\right)}^{2}\right]}^{1/2}$$

(2) $$H^{0}=\arctan\left(b^{*}/a^{*}\right)$$

### 2.5 Total anthocyanin concentration measurement

The TAC was determined according to the method described by Jiang and Nie (2015). Briefly, 1 ml of mulberry wine was added into two separate 10 ml volumetric flask containing two various buffer solutions with 0.2 mol/L KCl: 0.2 mol/L HCl = 25:67 (pH = 1) and 1 mol/L NaAc: 1 mol/L HCl: H_2_O = 100:60:90 (pH = 4.5), correspondingly with constant volume and kept in the dark for 2 hr. The absorbance of the two samples was measured at 510 nm and 700 nm (UV‐1600 spectrophotometer, Ruili Analytical Instrument, Beijing China), respectively. TAC was calculated according to Equations (3) and (4). The results were expressed as mg C3G/L.

(3) $$A={\left(A_{510}‐A_{700}\right)}_{\text{pH}1.0}‐{\left(A_{510}‐A_{700}\right)}_{\text{pH}4.5}$$

(4) $$\text{TAC}\left(\text{mgC}3G/L\right)=\frac{A\times \;\text{MW}\times \;\text{DF}\times 1000}{\varepsilon \times L}$$

Wherein *A* represents the absorbance as a result of Equation (3) and MW refers to molecular weight (C3G, MW = 449.2 mol/L), DF dilution factor (100), ɛ depicts the molar extinction co‐efficient (C3G, *ε* = 29,600 L/mol/cm), and *L* corresponds to optical path (1.0 cm).

### 2.6 Determination of the total phenolics concentration

A modified protocol of Zhang, Yang, et al. (2018) was adopted for TPC determination employing the Folin–Ciocalteu method. The absorbance of mulberry wine was measured at 760 nm with 1:10 dilution. The TPC of the sample was calculated from the gallic acid regression equation of the standard curve. The results were expressed in milligrams gallic acid equivalents (GAE) per liter (mg GAE/L).

### 2.7 Determination of the total flavonoids concentration

The TFC was determined by Sicari's method (Sicari et al., 2016) with minor modifications. Briefly, 1 ml of sample was poured into 50 ml volumetric flask containing 20 ml of 70% (v/v) ethanol. Then, 2 ml of NaNO_2_ solution (5%) was added and allowed to stand for 6 min. Consequently, the mixture was added with 2ml of Al(NO_3_)_3_ solution (10%) with continuous stirring and left to stand for 6 min. After that, 4 ml of 1.0 mol/L NaOH was added and made up with distilled water. After incubating at 25°C for 15 min, the absorbance was measured at 510 nm. The results were expressed in milligrams rutin equivalents (RE) per liter (mg RE/L). The standard curve for rutin was A = 0.0112*X* + 0.0008 (*R* ^2^ = 0.9998). *A* and *X* represent absorbance and concentration, respectively.

### 2.8 Determination of the antioxidant activity

#### 2.8.1 Determination of 2,2‐diphenyl‐1‐picrylhydrazyl scavenging activity (DPPH‐SA)

DPPH scavenging activity was determined by a modified method of Ivanovic et al. (2013). Briefly, 0.2 ml of mulberry wine at various concentrations (10, 30, 60, 100, 150, and 200 mg/L) was added to 7.8 ml of 95% ethanolic DPPH (0.025 mg/ml) solution and mixed thoroughly. The solution mixture was incubated in a dark room for 30 min. The absorbance of the sample was measured at 517 nm by a UV‐Vis spectrophotometer (Ruili Analytical Instrument, Beijing China). The results were expressed as millimoles Trolox Equivalent (TE) per liter (mmol TE/L).

(5) $$\text{DPPH - SA}\%=\frac{\left(A_{\text{blank}}‐A_{\text{sample}}\right)}{A_{\text{blank}}}\times 100$$

*A*_blank_: Absorbance of DPPH and methanol; *A*_sample_: Absorbance of sample and DPPH.

#### 2.8.2 2,2'‐azino‐bis (3‐ethylbenzothiazoline‐6‐sulfonic acid) scavenging activity (ABTS‐SA) measurement

ABTS‐SA of the mulberry fruit wine was evaluated by the ABTS cation decolorization assay with basic modifications (Tao et al., 2016). The ABTS radical cation (ABTS^+^) was generated by reaction of ABTS solution (7 mM) with potassium persulfate (2.45 mM). The reaction mixture was kept in the dark for 12 h at ambient temperature. The ABTS^+^ solution was mixed with ethanol to attain an optical density of 0.70 ± 0.02 at 734 nm. Briefly, 0.2 ml of mulberry wine at different concentrations (10, 30, 60, 100, 150, and 200 mg/L) was mixed with ABTS solution and the reaction mixture after 6 min was measured at 734 nm using a UV‐Vis spectrophotometer. The results were calculated as per Equation (6) and expressed in mmol TE/L.

(6) $$\text{ABTS - SA}\%=\frac{\left(A_{\text{blank}}‐A_{\text{sample}}\right)}{A_{\text{blank}}}\times 100$$

*A*_blank_: Absorbance of ABTS^+^ and methanol; *A*_sample_: Absorbance of sample and ABTS^+^.

#### 2.8.3 Determination of potassium ferricyanide reducing activity (PFRA)

The PFRA was performed with some modifications (Fang et al., 2011). The absorbance of the mulberry fruit wine at the various concentration (10, 30, 60, 100, 150, and 200 mg/L) was measured at 700 nm by a UV‐Vis spectrophotometer. The absorbance of the reaction mixture indicated the antioxidant activity of the sample, with a larger absorbance value indicating stronger antioxidant ability. The experimental results were expressed as millimoles ascorbic acid equivalent (AAE) per liter (mmol AAE/L).

### 2.9 Determination of phenolic acid content

The phenolic acid content of the mulberry wine was determined by the methods of Kwaw, Ma, Tchabo, Aapaliya, Wu, et al. (2018) using Shimadzu LC‐20AD (Kyoto, Japan). The phenolic acid content in the sample was calculated by a regression equation of 10 kinds of phenolic acid. The samples were filtered through a 0.45 μm microporous membrane and stored in the dark at 4°C.

Gradient elution: mobile phase A: methanol, acetic acid, water (10:2:88), mobile phase B: methanol, acetic acid, water (90:2:8), flow rate 1 ml/min.

Elution procedure: 0 to 25 min, B 0% to 10%; 25 to 45 min, B 10% to 50%; 45 to 53 min, B 50% to 0%; 53 to 58 min, B 0%. Detection wavelength: 280 nm.

### 2.10 Determination of taste quality

Kobayashi's method was used for the taste quality determination of the samples (Kobayashi et al., 2010). Precisely, the samples (diluted 5 times) were tested on the SA 402B Electronic Tongue (Insent, Japan). The fermented samples collected on the day 0 were taken as internal standards. The difference between the taste intensity values of other fermentation days and the internal standard was the relative intensity value. The intensity values of the internal standard were classified as 0.

### 2.11 Determination of aromatic components

The method of Tchabo was used for headspace solid‐phase microextraction (HS‐SPME) and GC‐MS (Agilent 6890/5973, Palo Alto, USA) parameters determination with trivial modifications (Tchabo, Ma, Kwaw, Zhang, Xiao, et al., 2017).

#### 2.11.1 Solid‐phase microextraction

Briefly, 5 ml of sample was added with 1.0 g NaCl, and internal standard was loaded into 15 ml of headspace bottle and preheated at 40°C for 10 min. Afterward, the extraction fiber (DVB/CAR/PDMS 50/30 µm, Supelco, USA) was inserted into the headspace bottle for 30 min, and the magnetic stirring speed was set at 800 rpm/min.

#### 2.11.2 GC‐MS parameter

Chromatographic conditions: chromatographic column DB‐WAX (60 m × 0.25 mm × 0.25 μm), extraction head resolution for 5 min, inlet temperature 250°C, carrier gas (He) flow rate 1.0 ml/min, no shunt injection. Programmed heating: kept at 50°C for 2 min, increased to 150°C at 6°C /min, held for 2 min, then raised to 220°C at 8°C/min, held for 7 min. Mass Spectrometry Conditions: 5973 Quadrupole Mass Spectrometer, Interface Temperature 250°C, Electron Bombardment (EI) Ion Source, Electronic Energy 70 eV, Electron Multiplier Voltage 1353 V, Ion Source Temperature 230°C, Quadrupole Temperature 150°C, Mass Scanning Range 33–450 amu.

#### 2.11.3 Qualitative and quantitative methods for volatile aroma components

Qualitative Methods encompass the data retrieval from spectral library retrieval, and components classification, corroboratively (Kwaw, Ma, Tchabo, Sackey, Apaliya, et al., 2018). The retention time of C_7_ to C_40_ mixtures of n‐alkanes proceeded for heating procedure as reported earlier and the retention index (RI) of aroma components was calculated according to the retention index formula (7).

(7) $$\text{RI}=100n+100\times \left(\frac{T‐T_{n}}{T_{n+1}‐T_{n}}\right)$$

RI refers to the retention index, and *n* and *n* + 1 are the number of carbon atoms of n‐alkanes before and after the outflow of the substance to be measured; *T_n_* and *T_n_*_+1_ represent the retention time of corresponding n‐alkanes; *T* is the retention time of the substance to be measured (*T_n_* < *T* < *T_n_*_+1_).

Quantitative analysis was carried out by internal standard method using 0.02 μl/ml n‐propanol). The concentration of the components was measured according to formula (8).

(8) $$C=\frac{A\times C_{i}}{A_{i}}$$

*C* corresponds to the concentration of the component and *A* refers to the peak area of the component; *C_i_* accounts for the concentration of the internal standard and the peak area of the internal standard depicts *A_i_*.

#### 2.11.4 Data processing and analysis

All the experiments were performed in triplicate, and the data were statistically represented as Mean ± Standard Deviation. The SPSS 17.0 (IBM, USA) was used for profiling significant differences between parametric variations. Origin Pro 2016 (OriginLab, USA) was used for computing the analysis of variance and drawing graphs with statistical inferences.

## 3 RESULTS AND DISCUSSION

### 3.1 Changes of color in mulberry wine during fermentation

**FIGURE 1:**
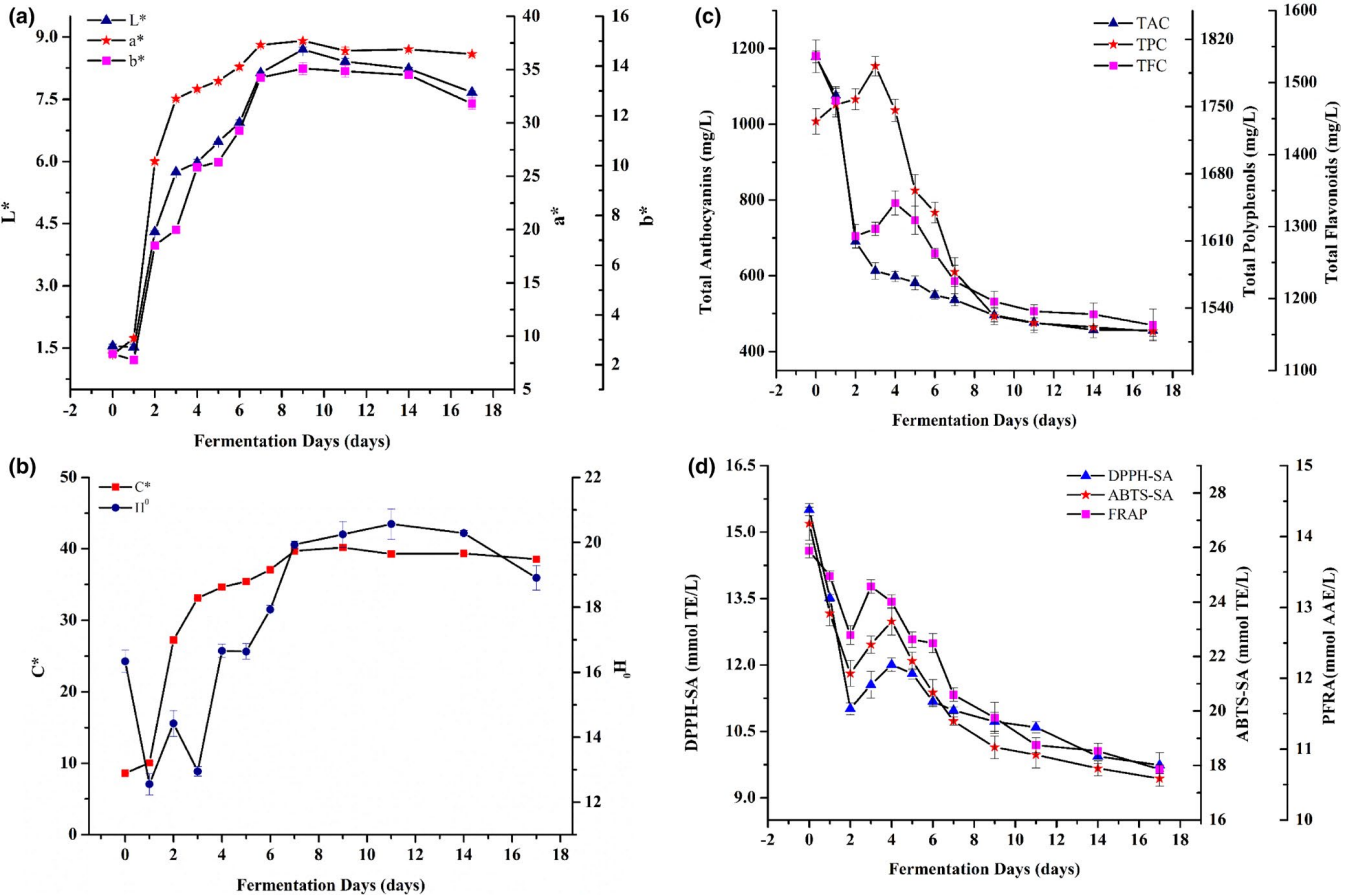
Changes of color (a and b), phenol concentration (c), and antioxidant capacity (d) during mulberry wine fermentation

Wine color changes in the mulberry wine samples are pictorially illustrated in Figure 1a, b. The results demonstrated that the transition pattern of the four‐color parameters (L*, a*, b*, and C*) was comparatively prominent. However, from 0 to day 1 of fermentation, the transition pattern of L* and b* was inversely proportional to that of a* and C*. The comparison of the four‐color parameters and TAC (Figure 1c) from day 1 to 9 revealed that the transition pattern of the four‐color parameters (L*, a*, b*, and C*) was opposite to TAC. Table 1 clearly revealed the significant inverse relationship between the four‐color parameters and TAC. From day 9 to 17, L* decreased significantly (*p* < .05), while a*, b*, and C* showed a slight fluctuation. This contradicted with the findings of Wang, Sun, et al. (2015) which could be attributed to the various varieties of mulberry fruits. The four‐color parameters increased gradually and then decreased during the fermentation period, and each color parameter increased dramatically after the fermentation period (*p* < .05). *H* ^0^ and C* increased significantly during the fermentation period (*p* < .05). The trend of *H* ^0^ change was consistent with that of L* from day 3 to 17, shown by a rapid rise followed by a decrease. According to earlier studies (Kwaw, Ma, Tchabo, Aapaliya, Wu, et al., 2018; Wang, Sun, et al., 2015), the change of L* and *H* ^0^ is closely related to TAC which shows negative correlation between TAC and L* and *H* ^0^, respectively.

**TABLE 1:** Pearson's correlation analysis between color and phenolics during the fermentation of mulberry wine

	L*	a*	b*	C*	*H* ^0^	TPC	TFC	TAC
L*	1							
a*	0.965**	1						
b*	0.991**	0.942**	1					
C*	0.974**	0.999**	0.954**	1				
*H* ^0^	0.796**	0.644*	0.852**	0.671*	1			
TPC	−0.786**	−0.620*	−0.827**	−0.647*	−0.920**	1		
TFC	−0.943**	−0.929**	−0.930**	−0.935**	−0.688*	0.759**	1	
TAC	−0.955**	−0.990**	−0.934**	−0.989**	−0.627*	0.641*	0.960**	1

Note

** means the correlation is extremely significant (*p* < .01); * means the correlation is significant (0.01 < *p* < .05).

### 3.2 Changes of TPC, TFC, and TAC in mulberry wine during fermentation and correlation analysis

The changes of TPC, TAC, and TFC in mulberry wine during fermentation are depicted in Figure 1c. The TPC increased from day 0 to 3 and reached the maximum (1792.17 mg GAE/L) on day 3. From day 3 to 9, the TPC decreased significantly (*p* < .05), and the decreasing rate was greater than the increasing rate at the start of fermentation. After the 9 days of fermentation, there was no significant difference (*p* > .05) between the TPC of the samples. TFC significantly decreased (*p* < .05) from day 0 to 2, and the TFC and TAC transition trends were similarly observed. Anthocyanin poses as an essential component of flavonoids, and hence, the depletion of TFC is closely linked to the deterioration of anthocyanins (Garrido & Borges, 2013). From day 2 to 4, the TFC increased, indicating the contents were still significantly lower than the initial quantity (*p* < .05). After 4 days, TFC decreased gradually. At the end of the fermentation period, the TPC and TFC of the samples were significantly decreased (*p* < .05) showing similar trends.

Anthocyanin is an important active component of mulberry, a kind of natural antioxidant, and has a positive effect on the human body with nutraceutical benefits (Mangani et al., 2011; Zhang et al., 2014). TAC significantly decreased (*p* < .05) from day 0 to 3 with a loss rating of 47.98% (Figure 1c). The decrease was great from day 1 to 2 at a loss of 32.77%. Overall, the TAC decreased rapidly and then tended to be stable during the fermentation process. Anthocyanins are susceptible to pH, temperature, sulfur dioxide, metal ions, enzymes, and ascorbic acid (Lopes et al., 2007). Studies have shown that yeast quantity and metabolites can affect anthocyanins (Mangani et al., 2011). Yeast multiplication and propagation result in rapid decomposition and culmination of anthocyanins. Also, anthocyanins efficiently reacted with o‐quinones produced by the oxidation of phenolic enzymes (Romero‐Cascales et al., 2005). All these could be the cause of the decline observed in the TAC of the samples. In general, the TAC significantly decreased (*p* < .05) after the fermentation period.

The significant positive correlation (*p* < .01) observed between the four‐color parameters (L*, a*, b*, and C*) is represented in Table 1. The results indicated that the strongest correlation was performed between a* and C* (*R* ^2^ = 0.999), indicating the escalated anthocyanin contents as evident from purple‐red coloration. The TPC had a significant negative correlation with L*, b*, and *H^0^* (*p* < .01). TFC and TAC were negatively correlated with L*, a*, b*, and C* (*p* < .01). Among phenolic compounds, TAC had the highest negative correlation with these four parameters (L*, a*, b*, and C*). The TAC was negatively correlated to the *H* ^0^ (*p* < .05), indicating that the change of *H^0^* was associated with the TAC establishing consistency (Kwaw, Ma, Tchabo, Sackey, Apaliya, et al., 2018). Similarly, the TPC had a significant negative correlation with *H^0^* (*R* ^2^ = −0.920). The trend of *H^0^* did not increase with the decrease of TAC in the early stage of the fermentation. This was due to the increase of TPC and the instability of the fermentation system.

### 3.3 Changes of antioxidant capacity during mulberry wine fermentation and correlation analysis

All the antioxidant activities measured by the three different methods decreased significantly (*p* < .05) from day 0 to 2, depicting the degradation and transformation of phenolics in initial stage of the fermentation (Figure 1d). Nevertheless, from day 2 to 4, DPPH‐SA and ABTS‐SA levels increased significantly (*p* < .05). However, PFRA increased significantly (*p* < .05) from day 2 to 3 and then decreased significantly (*p* < .05) from day 3 to 4. The increase in the activities of the three antioxidants observed may be attributed to the breakdown of further phenolic compounds in the ethanol generated by fermentation. The three antioxidant activities decreased as phenolic compounds decreased during the fourth day of fermentation. After the fermentation period, DPPH‐SA, ABTS‐SA, and PFRA levels were significantly lower than on day 0 (*p* < .05). Our findings differed from the report of Wang, Sun, et al. (2015) and Wang, Xie, et al. (2015), which may be attributed to differences in the fermentation mechanism, yeast species, and mulberry varieties used.

From Table 2, the antioxidant activity was highly correlated with TPC, TFC, and TAC. Among the phenolic compounds, an extremely significant correlation was observed between TPC and ABTS‐SA as well as TPC and PFRA (*p* < .01). The correlation between TAC, TFC, and the three antioxidant abilities was extremely significant. The strongest correlations were found between TPC and PFRA (*R* ^2^ = 0.939), TFC and DPPH‐SA (*R* ^2^ = 0.973), and TFC and ABTS‐SA (*R* ^2^ = 0.957). Antioxidant potentials of phenolic ingredients may be coerced to structural associations of simple glycosidic ligands and necessary regulation for quenching reactive oxygen species (Pérez‐Gregorio et al., 2011). The amount of acidity and phenolic hydroxyl can influence the relationship between flavonoid, phenols, and antioxidant efficacy. The resonance between the aromatic benzene ring and the phenoxy free electron pair enhances electron delocalization, promoting antioxidant activity against free radicals (Kwaw, Ma, Tchabo, Aapaliya, Wu, et al., 2018).

**TABLE 2:** Pearson's correlation analysis between phenolics and antioxidant capacity in the fermentation of mulberry

	DPPH‐SA	ABTS‐SA	PFRA	TPC	TFC	TAC
DPPH‐SA	1					
ABTS‐SA	0.941**	1				
PFRA	0.843**	0.957**	1			
TPC	0.654*	0.849**	0.939**	1		
TFC	0.973**	0.957**	0.897**	0.759**	1	
TAC	0.939**	0.860**	0.780**	0.641*	0.960**	1

Note

** means the correlation is extremely significant (*p* < .01); * means the correlation is significant (0.01 < *p* < .05).

### 3.4 Changes of phenolic acid content in mulberry wine during fermentation

**FIGURE 2:**
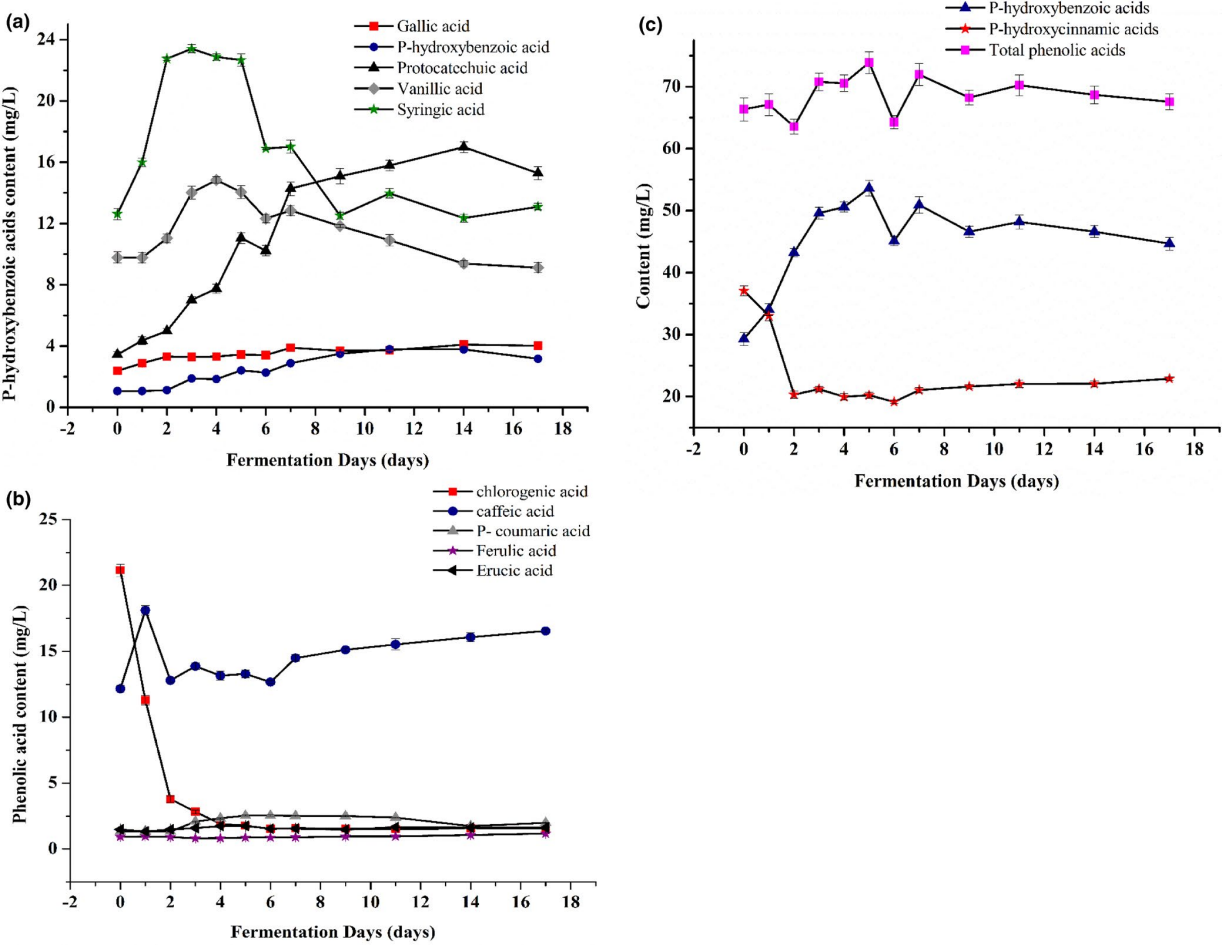
Changes of p‐hydroxybenzoic acids (a), p‐hydroxycinnamic acids (b), and total phenolic acid (c) during mulberry wine fermentation

The content of phenolic acid in the mulberry wine at different fermentation times is illustrated in Figure 2a, b. The most abundant phenolic acid constituent in the mulberry juice (fermentation 0th day) was chlorogenic acid (21.155 mg/L), which is consistent with the results reported by Gecer et al. (2016); however, there was a difference in content. The activities of glucosidase produced by *L. plantarum* resulted in the metabolism and release of the phenolic compounds (Zhou et al., 2020), leading to the significant (*p* < .01) increment observed in the content of protocatechuic acid. Research proved that *Lactiplantibacillus* metabolizes catechin and gallic acid to protocatechuic acid (Valero‐Cases et al., 2017). The contents of vanillic acid, syringic acid, and *p*‐coumaric acid first increased and then decreased, and optimally saturated on the days 4, 3, and 5, respectively. Chlorogenic acid reduced dramatically from day 0 to 2 and then stabilized, consequently. LAB can hydrolyze chlorogenic acid to release caffeic acid (Fritsch et al., 2016). However, the content of caffeic acid initially increased, then decreased, and reached the maximum (18.115 mg/L) on day 1. During the fermentation period, the levels of ferulic acid and erucic acid slightly fluctuated.

The transition pattern of total *p*‐hydroxybenzoic acids (gallic acid, *p*‐hydroxybenzoic acid, protocatechuic acid, vanillic acid, and syringic acid), *p*‐hydroxycinnamic acids (chlorogenic acid, caffeic acid, *p*‐coumarinic acid, ferulic acid, and erucic acid), and phenolic acid content was analyzed to better understand the overall change of phenolic acid throughout the fermentation phase. The transition pattern is depicted in Figure 2c. The total phenolic acid content was dynamically balanced and maintained at an enhanced level. From day 0 to 2, the content of total *p*‐hydroxybenzoic acids increased by 13.936 mg/L while the content of *p*‐hydroxycinnamic acids decreased by 16.727 mg/L. Chlorogenic acid and ferulic acid levels decreased by 17.357 mg/L and 0.016 mg/L, respectively, suggesting that chlorogenic acid was the main cause attributed for decreased levels of *p*‐hydroxycinnamic acids from day 0 to 2 of fermentation. *p*‐Hydroxycinnamic acid production was reduced due to the increased levels of caffeic acid, *p*‐coumaric acid, and erucic acid which was less than the decrease of chlorogenic acid and ferulic acid. The composition of *p*‐hydroxybenzoic acids and overall phenolic acid showed similar pattern changes after day 2 of fermentation. Hence, *p*‐hydroxybenzoic acids are referred for content variations in total phenolic acid contents.

### 3.5 Taste and quality changes of mulberry wine during fermentation

**FIGURE 3:**
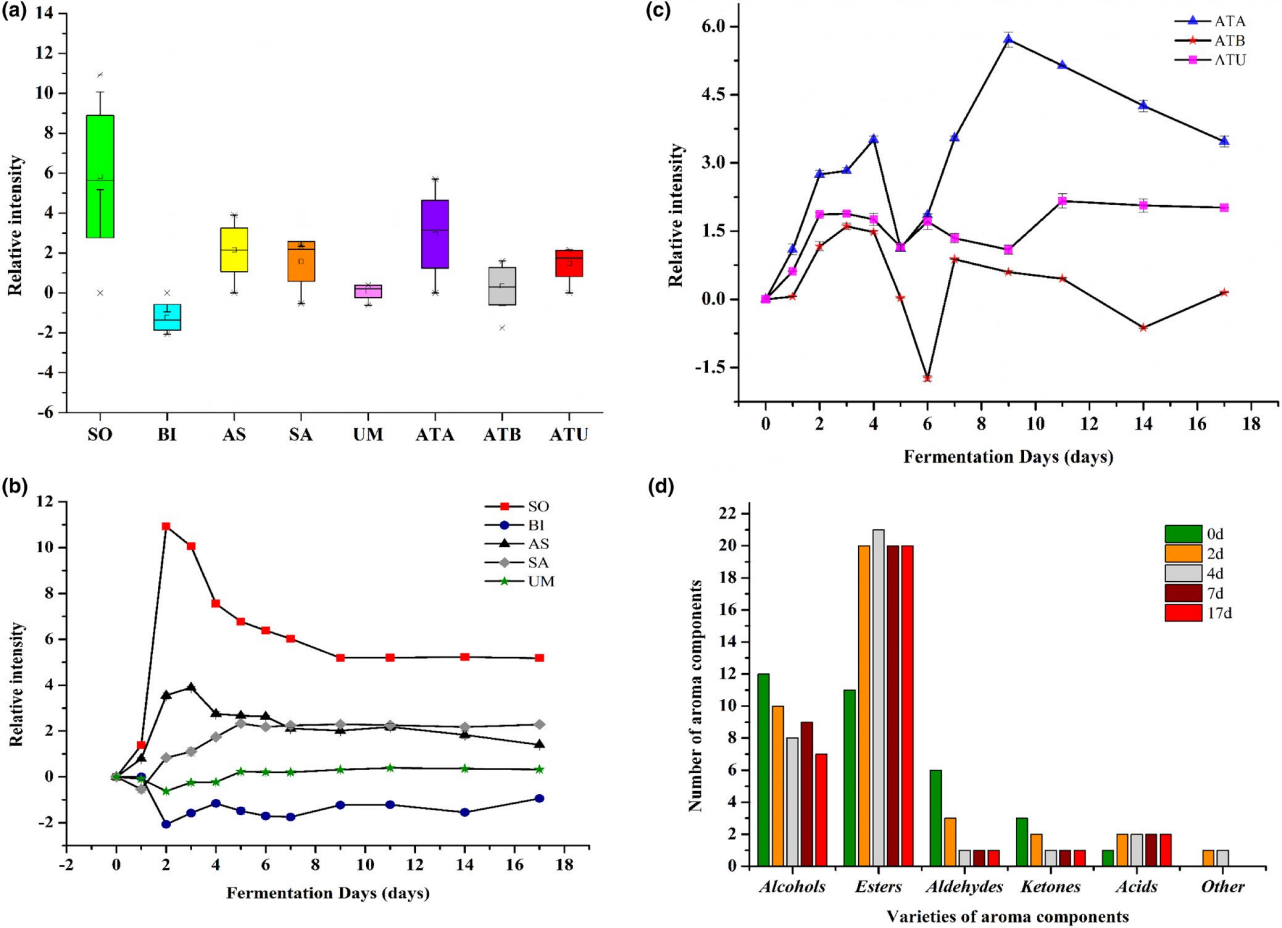
The box plot of relative intensity of each taste index (a), the relative intensity of each taste index (b and c), varieties of aroma components in mulberry wine fermentation (d); SO‐Sour, BI‐Bitter, AS‐Astringent, SA‐Salty, UM‐Umami, ATA‐Aftertaste of Astringent, ATB‐ Aftertaste of Bitter, and ATU‐Aftertaste of Umami

Wine taste was detected using the detection sensor of the electronic tongue which comprise an artificial lipid membranous cast in an electrode widely used for the routine assessment (Lu et al., 2016). Twelve mulberry wine samples with different fermentation times were investigated to determine the changing trend of five basic flavors (acid, bitter, astringent, salty, and umami) and the aftertaste of bitter (ATB), astringent (ATA), and umami (ATU). According to Figure 3a, the relative intensity of each flavor index of the 12 mulberry wine samples varied significantly, with the sourness index demonstrating a tremendous variation (10.93). However, the umami was 1.01 (the lowest). According to Kobayashi et al. (2010), if the difference of the relative strength of two samples on the same index is greater than 1, in the cause of measuring and evaluating their taste index with electronic tongue, then the difference can be identified by sensory evaluation. Hence, the changes in taste indexes of mulberry wine could be distinguished by sensory evaluation.

Figure 3b,c showed that sourness reached a maximum of 10.93) on day 2. However, from day 9 to day 17, stability in taste was established. The sourness was reflected by the pH of the wine rendered through fermentation efficacy. During the early stages of fermentation, the yeast grew and generated many acidic metabolites and CO_2_, subsequently making the pH to drop dramatically and the fermentation broth sour. The relative intensity of bitterness reached the minimum (−2.07) on day 2. However, astringency first increased, then decreased significantly (*p* < .05), and reached the maximum (3.90) on day 3. The bitterness and astringency of wine are mostly due to phenolic compounds (Cai et al., 2020). The phenolic compounds in mulberry wine steadily decreased as fermentation continues, resulting in a steady decrease in astringency and bitterness. From day 0 to 5, the relative intensity of saltiness and umami declined, subsequently increasing the significance (*p* < .05), reaching a minimum on day 1 (−0.54) and day 2 (−0.62), respectively. Due to alcohol fermentation, the relative strength of the five simple flavors changed dramatically from day 0 to 4 and the substrates were stabilized after 4th day. From day 0 to 4, ATA, ATB, and ATU demonstrated an average upward trending comprehensively. From day 4 to 17, ATA and ATB fluctuated significantly with initial decrease and drastic variations except for ATU that remained stable. According to our findings, the taste of mulberry wine samples became more durable, and unpleasant flavors diminished.

### 3.6 Changes of aroma components in mulberry wine during fermentation

The mulberry wine samples were harvested and analyzed on days 0, 2, 4, 7, and 17. It showed the quantity and content of aroma components in Figure 3d and Table 3. In mulberry wine, 51 volatile substances were detected by using SPME‐GC‐MS method, including 13 alcohols, 24 esters, seven aldehydes, three ketones, three acids, and one aromatics component. The results indicated that the total volatile components content first increased and then decreased, and reached the maximum (11,808.48 μg/L) on the 7th day. Furthermore, after fermentation, the total volatile compounds of mulberry wine were significantly (*p* < .05) higher than mulberry juice (fermentation 0th day). As a whole fermentation process, alcohols and esters accounted for the largest proportion of the total volatile components constituents.

**TABLE 3:** Changes of aromatic components during the fermentation of mulberry wine

Number	RT (Min)	Compound	RI (RIExp/RIL)	Content (μg/L)				
				0 days	2 days	4 days	7 days	17 days
*Alcohol*								
1	9.24	Ethanol	923/922	32.38 ± 0.29	415.34 ± 6.21	1848.87 ± 23.41	3184.91 ± 17.39	1919.59 ± 27.31
2	12.87	Isobutanol	1079/1080	9.08 ± 0.31	124.34 ± 2.63	247.87 ± 2.89	336.42 ± 0.54	267.97 ± 1.47
3	14.10	*N*‐butanol	1127/1137	3.89 ± 0.20	2.46 ± 0.37	6.55 ± 0.40	7.56 ± 0.66	5.71 ± 0.21
4	15.70	Isoamyl alcohol	1189/1187	–	313.61 ± 7.45	754.89 ± 4.58	1172.30 ± 12.64	955.06 ± 13.06
5	16.70	Amyl alcohol	1229/1231	1.82 ± 0.13	–	–	–	–
6	19.14	Hexanol	1328/1340	16.42 ± 0.73	1.80 ± 0.14	–	–	–
7	21.57	Heptanol	1429/1450	1.72 ± 0.05	–	12.40 ± 0.23	14.56 ± 0.09	13.34 ± 0.33
8	22.45	2‐ethylhexanol	1463/1462	1.06 ± 0.11	–	–	–	–
9	24.22	1‐octanol	1534/1539	1.34 ± 0.09	3.50 ± 0.11	–	5.89 ± 0.12	3.67 ± 0.05
10	24.52	2,3‐butanediol	1546/1554	3.98 ± 0.29	3.42 ± 0.14	10.91 ± 0.23	11.26 ± 0.33	–
11	25.75	4‐nonenol	1596/1569	2.98 ± 0.05	2.66 ± 0.19	6.96 ± 0.20	–	–
12	26.65	1‐nonanol	1636/1637	2.00 ± 0.16	4.35 ± 0.23	–	11.07 ± 0.10	–
13	31.69	Phenylethanol	1895/1906	3.87 ± 0.03	69.32 ± 1.11	158.06 ± 5.40	256.34 ± 1.66	168.51 ± 1.19
				80.53 ± 1.55^e^	940.81 ± 12.00^d^	3046.51 ± 26.20^c^	5000.32 ± 24.51^a^	3333.85 ± 26.39^b^
*Ester*								
14	7.59	Methyl acetate	821/826	15.50 ± 0.38	3.43 ± 0.25	19.20 ± 0.13	10.84 ± 0.21	9.43 ± 0.22
15	8.45	Ethyl acetate	879/883	194.55 ± 1.12	166.00 ± 0.99	300.04 ± 2.06	476.35 ± 1.08	471.29 ± 1.78
16	9.82	Ethyl propionate	950/953	2.03 ± 0.20	5.04 ± 0.51	7.68 ± 0.16	6.45 ± 0.21	4.23 ± 0.20
17	10.00	Ethyl isobutyrate	958/937	1.38 ± 0.07	–	–	–	–
18	10.17	Propyl acetate	966/980	1.85 ± 0.05	4.01 ± 0.64	6.55 ± 0.54	6.94 ± 0.69	5.81 ± 0.14
19	10.41	Methyl butyrate	977/976	1.65 ± 0.24	–	–	–	–
20	11.01	Isobutyl acetate	1005/1006	1.80 ± 0.15	29.17 ± 0.75	75.11 ± 2.42	74.65 ± 0.94	66.83 ± 1.87
21	11.58	Ethyl butyrate	1027/1034	–	40.74 ± 1.07	49.63 ± 1.78	50.42 ± 0.95	46.07 ± 1.07
22	11.76	Propyl propionate	1035/1047	6.60 ± 0.27	–	–	–	–
23	11.96	Ethyl 2‐methylbutyrate	1043/1048	1.30 ± 0.07	10.00 ± 0.68	11.37 ± 0.44	9.37 ± 0.29	10.60 ± 0.27
24	13.82	Isoamyl acetate	1116/1113	–	453.37 ± 6.54	1171.86 ± 35.60	1324.96 ± 10.28	1084.66 ± 24.73
25	14.75	Ethyl crotonate	1152/1156	–	0.72 ± 0.16	2.47 ± 0.41	4.36 ± 0.32	3.77 ± 0.34
26	16.53	Ethyl hexanoate	1222/1227	1.23 ± 0.18	340.23 ± 9.82	283.12 ± 3.95	341.41 ± 18.26	317.05 ± 16.69
27	17.44	Hexyl acetate	1258/1268	–	12.15 ± 0.28	10.45 ± 0.23	12.39 ± 0.32	6.84 ± 0.52
28	19.03	Ethyl heptanoate	1324/1327	–	2.21 ± 0.13	2.50 ± 0.25	4.41 ± 0.22	4.77 ± 0.35
30	20.24	Methyl octanoate	1375/1389	5.86 ± 0.25	19.92 ± 1.59	28.65 ± 0.73	47.87 ± 1.62	40.76 ± 1.41
31	21.41	Ethyl octanoate	1422/1421	–	1132.68 ± 6.20	1937.43 ± 5.18	2721.32 ± 10.83	2411.61 ± 15.12
32	22.35	Octyl acetate	1459/1474	–	4.31 ± 0.12	10.48 ± 0.13	5.82 ± 0.17	–
33	23.97	Ethyl citrate	1524/1538	–	4.83 ± 0.20	11.26 ± 0.15	9.88 ± 0.36	6.29 ± 0.37
34	26.43	Ethyl citrate	1627/1622	–	261.10 ± 4.63	1798.27 ± 17.23	1074.42 ± 13.93	790.11 ± 9.08
35	27.35	Ethyl benzoate	1668/1673	–	–	4.28 ± 0.15	–	2.98 ± 0.15
36	30.12	Phenylacetate	1805/1801	–	87.80 ± 1.78	140.56 ± 2.90	197.84 ± 4.80	132.80 ± 2.09
37	30.56	Ethyl laurate	1830/1849	–	37.77 ± 1.03	198.45 ± 2.92	237.57 ± 3.55	63.77 ± 0.70
				233.76 ± 1.64^e^	2618.70 ± 4.90^d^	6074.02 ± 51.81^b^	6621.59 ± 27.77^a^	5482.69 ± 19.69^c^
*Aldehyde*								
38	6.42	Acetaldehyde	715/716	4.35 ± 0.16	5.30 ± 0.20	6.81 ± 0.11	10.52 ± 0.34	9.07 ± 0.32
39	8.98	2‐methylbutanal	910/907	3.28 ± 0.23	–	–	–	–
40	9.05	Isovaleraldehyde	914/913	5.31 ± 0.09	2.61 ± 0.39	–	–	–
41	12.71	Hexanal	1073/1077	7.48 ± 0.45	–	–	–	–
42	16.22	2‐hexenal	1209/1212	1.30 ± 0.14	–	–	–	–
43	23.10	Furfural	1489/1504	–	1.98 ± 0.12	–	–	–
44	23.88	Benzaldehyde	1520/1525	1.66 ± 0.04	–	–	–	–
				23.39 ± 0.53^a^	9.88 ± 0.42^b^	6.81 ± 0.11^d^	10.52 ± 0.34^b^	9.07 ± 0.32^c^
*Ketones*								
45	10.08	2,3‐butanedione	962/952	2.51 ± 0.25	2.27 ± 0.19	6.20 ± 0.24	13.54 ± 0.61	16.45 ± 0.36
46	15.27	2‐heptanone	1172/1185	1.24 ± 0.12	–	–	–	–
47	17.79	3‐hydroxy‐2‐butanone	1273/1287	15.36 ± 0.10	2.12 ± 0.04	–	–	–
				19.11 ± 0.41^a^	4.38 ± 0.16^e^	6.20 ± 0.24^d^	13.54 ± 0.61^c^	16.45 ± 0.36^b^
Acid								
48	21.27	Acetic acid	1417/1405	33.70 ± 0.41	–	–	–	–
49	30.21	Caproic acid	1810/1807	–	11.16 ± 0.38	12.34 ± 0.07	25.25 ± 0.27	19.40 ± 0.53
50	33.89	Octanoic acid	2022/2051	–	90.74 ± 1.19	92.50 ± 0.69	137.26 ± 0.95	96.79 ± 1.87
				33.70 ± 0.41^e^	101.90 ± 0.84^d^	104.84 ± 0.63^c^	162.51 ± 0.73^a^	116.20 ± 1.51^b^
*Other*								
51	17.19	Styrene	1248/1259	–	4.80 ± 0.26	4.17 ± 0.23	–	–
		Total		390.49 ± 4.54^e^	3680.47 ± 18.58^d^	9242.55 ± 79.22^b^	11,808.48 ± 53.96^a^	8958.26 ± 48.27^c^

Abbreviations: RIExp, Retention Index Experiment; RIL, Retention Index Library;"–": Not detected. The superscript alphabetical letters a, b, c, d, and e denote statistically significant differences between the data (*p* < 0.05).

According to Belda et al. (2017), alcohols are essential flavor compounds in fruit wine, and they are mostly generated through glycolysis. Furthermore, amino acids precursors in resultant higher alcoholic compositions play a pivotal role. Among the identified volatile compounds, alcohols played an important role in volatile substances, accounting for 20.62% to 42.37% of the total volatile substances. Ethanol, isobutanol, isoamyl alcohol, and phenylethanol were the principal components of alcohols, and their levels reached the maximum on the 7th day. Isoamyl alcohol presented the greatest concentration of higher alcohols in mulberry wine. Additionally, phenylethanol was found to impart a distinct floral fragrance to mulberry wine (Wang, Xie, et al., 2015). Compared with mulberry juice (fermentation 0th day), the amount of alcohol in mulberry wine after day 17 of fermentation decreased. However, the overall content was significantly higher than that in mulberry juice (*p* < .05). The content of alcohol group increased from 80.53 μg/L to 3333.85 μg/L after fermentation. Hexanol (16.42 μg/L) was the most important alcohol volatile substance in mulberry juice, which has a grassy and woody fragrance, decreased rapidly during the fermentation period. However, it was not detected on the 17th day of fermentation. After fermentation, isoamyl alcohol was the main alcohol volatile in mulberry wine which was produced during the fermented process.

As the largest family of mulberry wine volatile compounds, esters accounted for 56.07% to 71.15% of the total identified compounds. It has been proposed that esters are formed by the reaction of alcohols and organic acids during the fermentation process, which has a significant impact on the aroma of wine (Belda et al., 2017). Furthermore, the presence of yeast and other microbes might result in the production of esters (Wang, Xie, et al., 2015). Among the volatile esters identified, ethyl acetate, isoamyl acetate, ethyl hexanoate, ethyl octanoate, and ethyl citrate predominated in ester family. Ethyl caprylate presented the highest ester content during the fermentation process, but was not detected in mulberry juice, reaching a maximum of 2721.32 μg/L after day 7. The research suggested that ethyl acetate has a significant effect on the fruity aroma of the wine. Isobutyl acetate has a floral fragrance and is produced by the reaction of isobutanol and acetic acid in fruit wine. Moreover, isoamyl acetate has a strong aroma similar to banana (Peng et al., 2016). Some esters disappeared at the early stage, however appeared with the progress of fermentation. In the comparison of the aroma at the end of the fermentation period (fermentation 17th day) to that at day 0 of fermentation, 9 ester aroma components were added, and the total esters content increased from 233.76 μg/L to 5482.69 μg/L. Moreover, the change of ethyl octanoate was the most significant after fermentation. Thus, the days of fermentation from 0 to 17 clearly depicts transesterification reactions with augmented ester contents.

## 4 CONCLUSIONS

The strains, *Lactiplantibacillus plantarum* and *Saccharomyces cerevisiae*, used for the fermentation influenced the phenolic compounds (TPC, TAC, and TFC) of the mulberry wine samples. During the fermentation period, the total anthocyanin, phenolic, and flavonoid content decreased significantly in the mulberry wine. Furthermore, mulberry wine exhibited better free radical quenching ability due to the presence of microbially biotransformed phytochemical compounds after *L*. *plantarum* and *S. cerevisiae* fermentation. These compounds influenced the quality of mulberry wine. Fermentation with LAB and yeast significantly influenced color, taste, aroma, phenolic profiles, and antioxidant properties of the mulberry wine samples, which could focus the application prospects for nutraceutical and healthcare potentials. Besides, mulberry wine production makes maximum use of mulberry fruits, prevents post‐harvest losses, and demonstrates significant economic potential.

## CONFLICT OF INTEREST

The authors have no conflict of interest to declare.

## AUTHOR CONTRIBUTIONS

**Jie Hu:** Writing‐original draft (equal); Writing‐review & editing (equal). **Annadurai Vinothkanna:** Writing‐original draft (equal); Writing‐review & editing (equal). **Meng Wu:** Methodology (equal); Writing‐review & editing (equal). **John‐Nelson Ekumah:** Writing‐review & editing (equal). **Nelson Dzidzorgbe Kwaku Akpabli‐Tsigbe:** Writing‐review & editing (equal). **Yongkun Ma:** Conceptualization (equal); Project administration (equal).

## ETHICAL STATEMENT

This research article does not contain any studies with human participants or animals performed by any of the authors.

## References

[fsn32590-bib-0001] Bao, T. , Xu, Y. , Gowd, V. , Zhao, J. C. , Xie, J. H. , Liang, W. K. , & Chen, W. (2016). Systematic study on phytochemicals and antioxidant activity of some new and common mulberry cultivars in China. Journal of Functional Foods, 25, 537–547. 10.1016/j.jff.2016.07.001

[fsn32590-bib-0002] Belda, I. , Ruiz, J. , Esteban‐Fernandez, A. , Navascues, E. , Marquina, D. , Santos, A. , & Moreno‐Arribas, M. V. (2017). Microbial contribution to wine aroma and its intended use for wine quality improvement. Molecules, 22(2), 189–218. 10.3390/molecules22020189 PMC6155689 28125039

[fsn32590-bib-0003] Cai, W. , Tang, F. , Guo, Z. , Guo, X. , Zhang, Q. , Zhao, X. , Ning, M. , & Shan, C. (2020). Effects of pretreatment methods and leaching methods on jujube wine quality detected by electronic senses and HS‐SPME‐GC‐MS. Food Chemistry, 330(2020), 127330. 10.1016/j.foodchem.2020.127330. 32569941

[fsn32590-bib-0004] Fang, Y. , Meng, J. , Zhang, A. , Liu, J. , Xu, T. , Yu, W. , Chen, S. , Li, H. , Zhang, Z. , & Wang, H. (2011). Influence of shriveling on berry composition and antioxidant activity of Cabernet Sauvignon grapes from Shanxi vineyards. Journal of the Science of Food and Agriculture, 91(4), 749–757. 10.1002/jsfa.4246 21302331

[fsn32590-bib-0005] Feng, Y. , Liu, M. , Ouyang, Y. , Zhao, X. , Ju, Y. , & Fang, Y. (2015). Comparative study of aromatic compounds in fruit wines from raspberry, strawberry, and mulberry in central Shaanxi area. Food & Nutrition Research, 59(1), 29290. 10.3402/fnr.v59.29290 26617387 PMC4663194

[fsn32590-bib-0006] Fritsch, C. , Heinrich, V. , Vogel, R. F. , & Toelstede, S. (2016). Phenolic acid degradation potential and growth behavior of lactic acid bacteria in sunflower substrates. Food Microbiology, 57, 178–186. 10.1016/j.fm.2016.03.003 27052717

[fsn32590-bib-0007] Garrido, J. , & Borges, F. (2013). Wine and grape polyphenols — A chemical perspective. Food Research International, 54(2), 1844–1858. 10.1016/j.foodres.2013.08.002

[fsn32590-bib-0008] Gecer, M. K. , Akin, M. , Gundogdu, M. , Eyduran, S. P. , Ercisli, S. , & Eyduran, E. (2016). Organic acids, sugars, phenolic compounds, and some horticultural characteristics of black and white mulberry accessions from Eastern Anatolia. Canadian Journal of Plant Science, 96(1), 27–33. 10.1139/cjps-2015-0070

[fsn32590-bib-0009] Ivanovic, J. , Dimitrijevic‐Brankovic, S. , Misic, D. , Ristic, M. , & Zizovic, I. (2013). Evaluation and improvement of antioxidant and antibacterial activities of supercritical extracts from clove buds. Journal of Functional Foods, 5(1), 416–423. 10.1016/j.jff.2012.11.014

[fsn32590-bib-0010] Jiang, Y. , & Nie, W. J. (2015). Chemical properties in fruits of mulberry species from the Xinjiang province of China. Food Chemistry, 174, 460–466. 10.1016/j.foodchem.2014.11.083 25529706

[fsn32590-bib-0011] Juan, C. , Jianquan, K. , Junni, T. , Zijian, C. , & Ji, L. (2012). The profile in polyphenols and volatile compounds in alcoholic beverages from different cultivars of mulberry. Journal of Food Science, 77(4), C430–436. 10.1111/j.1750-3841.2011.02593.x 22352465

[fsn32590-bib-0012] Kavitha, Y. , & Geetha, A. (2018). Anti‐inflammatory and preventive activity of white mulberry root bark extract in an experimental model of pancreatitis. Journal of Traditional and Complementary Medicine, 8(4), 497–505. 10.1016/j.jtcme.2018.01.011 30302330 PMC6174261

[fsn32590-bib-0013] Khalifa, I. , Zhu, W. , Li, K. K. , & Li, C. M. (2018). Polyphenols of mulberry fruits as multifaceted compounds: Compositions, metabolism, health benefits, and stability—A structural review. Journal of Functional Foods, 40, 28–43. 10.1016/j.jff.2017.10.041

[fsn32590-bib-0014] Kobayashi, Y. , Habara, M. , Ikezazki, H. , Chen, R. , Naito, Y. , & Toko, K. (2010). Advanced taste sensors based on artificial lipids with global selectivity to basic taste qualities and high correlation to sensory scores. Sensors (Basel), 10(4), 3411–3443. 10.3390/s100403411 22319306 PMC3274227

[fsn32590-bib-0015] Kwaw, E. , Ma, Y. K. , Tchabo, W. , Apaliya, M. T. , Sackey, A. S. , Wu, M. , & Xiao, L. L. (2018). Effect of pulsed light treatment on the phytochemical, volatile, and sensorial attributes of lactic‐acid‐fermented mulberry juice. International Journal of Food Properties, 21(1), 228–243. 10.1080/10942912.2018.1446024

[fsn32590-bib-0016] Kwaw, E. , Ma, Y. , Tchabo, W. , Apaliya, M. T. , Wu, M. , Sackey, A. S. , Xiao, L. , & Tahir, H. E. (2018). Effect of *lactobacillus* strains on phenolic profile, color attributes and antioxidant activities of lactic‐acid‐fermented mulberry juice. Food Chemistry, 250, 148–154. 10.1016/j.foodchem.2018.01.009 29412905

[fsn32590-bib-0017] Kwaw, E. , Ma, Y. , Tchabo, W. , Sackey, A. S. , Apaliya, M. T. , Xiao, L. , Wu, M. , & Sarpong, F. (2018). Ultrasonication effects on the phytochemical, volatile and sensorial characteristics of lactic acid fermented mulberry juice. Food Bioscience, 24, 17–25. 10.1016/j.fbio.2018.05.004

[fsn32590-bib-0018] Li, F. , Zhang, B. , Chen, G. , & Fu, X. (2017). The novel contributors of anti‐diabetic potential in mulberry polyphenols revealed by UHPLC‐HR‐ESI‐TOF‐MS/MS. Food Research International, 100(Pt1), 873–884. 10.1016/j.foodres.2017.06.052 28873762

[fsn32590-bib-0019] Liu, S. J. , Wu, C. E. , Fan, G. J. , Li, T. T. , Ying, R. F. , & Miao, Y. (2017). Effects of yeast strain on anthocyanin, color, and antioxidant activity of mulberry wines. Journal of Food Biochemistry, 41(6), e12409. 10.1111/jfbc.12409

[fsn32590-bib-0020] Lopes, P. , Richard, T. , Saucier, C. , Teissedre, P. L. , Monti, J. P. , & Glories, Y. (2007). Anthocyanone A: A quinone methide derivative resulting from malvidin 3‐O‐glucoside degradation. Journal of Agricultural and Food Chemistry, 55(7), 2698–2704. 10.1021/jf062875o 17338545

[fsn32590-bib-0021] Lu, L. , Hu, X. , Tian, S. , Deng, S. , & Zhu, Z. (2016). Visualized attribute analysis approach for characterization and quantification of rice taste flavor using electronic tongue. Analytica Chimica Acta, 919, 11–19. 10.1016/j.aca.2016.03.019 27086094

[fsn32590-bib-0022] Mahboubi, M. (2019). *Morus* *alba* (mulberry), a natural potent compound in management of obesity. Pharmacological Research, 146, 104341. 10.1016/j.phrs.2019.104341 31276774

[fsn32590-bib-0023] Mangani, S. , Buscioni, G. , Collina, L. , Bocci, E. , & Vincenzini, M. (2011). Effects of microbial populations on anthocyanin profile of sangiovese wines produced in Tuscany, Italy. American Journal of Enology and Viticulture, 62(4), 487–494. 10.5344/ajev.2011.11047

[fsn32590-bib-0024] Peng, B. , Li, F. , Cui, L. , & Guo, Y. (2016). Effects of fermentation temperature on key aroma compounds and sensory properties of apple wine. Journal of Food Science, 80(12), S2937–S2943. 10.1111/1750-3841.13111 26509667

[fsn32590-bib-0025] Pérez‐Gregorio, M. R. , Regueiro, J. , Alonso‐González, E. , Pastrana‐Castro, L. M. , & Simal‐Gándara, J. (2011). Influence of alcoholic fermentation process on antioxidant activity and phenolic levels from mulberries (*Morus* *nigra* L.). LWT‐ Food Science and Technology, 44(8), 1793–1801. 10.1016/j.lwt.2011.03.007

[fsn32590-bib-0026] Romero‐Cascales, I. , Fernandez‐Fernandez, J. I. , Lopez‐Roca, J. M. , & Gomez‐Plaza, E. (2005). The maceration process during winemaking extraction of anthocyanins from grape skins into wine. European Food Research and Technology, 221(1–2), 163–167. 10.1007/s00217-005-1144-1

[fsn32590-bib-0027] Sanchez‐Salcedo, E. M. , Mena, P. , Garcia‐Viguera, C. , Martinez, J. J. , & Hernandez, F. (2015). Phytochemical evaluation of white (*Morus* *alba* L.) and black (*Morus* *nigra* L.) mulberry fruits, a starting point for the assessment of their beneficial properties. Journal of Functional Foods, 12, 399–408. 10.1016/j.jff.2014.12.010

[fsn32590-bib-0028] Sicari, V. , Pellicano, T. M. , Giuffre, A. M. , Zappia, C. , & Capocasale, M. (2016). Bioactive compounds and antioxidant activity of citrus juices produced from varieties cultivated in Calabria. Journal of Food Measurement and Characterization, 10(4), 773–780. 10.1007/s11694-016-9362-8

[fsn32590-bib-0029] Tao, Y. , Sun, D. W. , Gorecki, A. , Blaszczak, W. , Lamparski, G. , Amarowicz, R. , Fornal, J. , & Jelinski, T. (2016). A preliminary study about the influence of high hydrostatic pressure processing in parallel with oak chip maceration on the physicochemical and sensory properties of a young red wine. Food Chemistry, 194, 545–554. 10.1016/j.foodchem.2015.07.041 26471591

[fsn32590-bib-0030] Tchabo, W. , Ma, Y. K. , Kwaw, E. , Zhang, H. N. , & Li, X. (2017). Influence of fermentation parameters on phytochemical profile and volatile properties of mulberry (*Morus* *nigra*) wine. Journal of the Institute of Brewing, 123(1), 151–158. 10.1002/jib.401

[fsn32590-bib-0031] Tchabo, W. , Ma, Y. , Kwaw, E. , Zhang, H. , Xiao, L. , & Tahir, H. E. (2017). Aroma profile and sensory characteristics of a sulfur dioxide‐free mulberry (*Morus* *nigra*) wine subjected to non‐thermal accelerating aging techniques. Food Chemistry, 232, 89–97. 10.1016/j.foodchem.2017.03.160 28490149

[fsn32590-bib-0032] Valero‐Cases, E. , Nuncio‐Jauregui, N. , & Frutos, M. J. (2017). Influence of fermentation with different lactic acid bacteria and in vitro digestion on the biotransformation of phenolic compounds in fermented pomegranate juices. Journal of Agricultural and Food Chemistry, 65(31), 6488–6496. 10.1021/acs.jafc.6b04854 28274113

[fsn32590-bib-0033] Vasserot, Y. , Mornet, F. , & Jeandet, P. (2010). Acetic acid removal by *Saccharomyces cerevisiae* during fermentation in oenological conditions. Metabolic consequences. Food Chemistry, 119(3), 1220–1223. 10.1016/j.foodchem.2009.08.008

[fsn32590-bib-0034] Wang, L. H. , Sun, X. Y. , Li, F. , Yu, D. , Liu, X. Y. , Huang, W. D. , & Zhan, J. C. (2015). Dynamic changes in phenolic compounds, colour and antioxidant activity of mulberry wine during alcoholic fermentation. Journal of Functional Foods, 18, 254–265. 10.1016/j.jff.2015.07.013

[fsn32590-bib-0035] Wang, X. , Xie, K. , Zhuang, H. , Ye, R. , Fang, Z. , & Feng, T. (2015). Volatile flavor compounds, total polyphenolic contents and antioxidant activities of a China gingko wine. Food Chemistry, 182, 41–46. 10.1016/j.foodchem.2015.02.120 25842306

[fsn32590-bib-0036] Wei, H. , Liu, S. , Liao, Y. , Ma, C. , Wang, D. , Tong, J. , Feng, J. , Yi, T. , & Zhu, L. (2018). A systematic review of the medicinal potential of mulberry in treating diabetes mellitus. American Journal of Chinese Medicine, 46(8), 1743–1770. 10.1142/S0192415X1850088X 30518235

[fsn32590-bib-0037] You, Y. L. , Li, N. , Han, X. , Guo, J. L. , Zhao, Y. , Liu, G. J. , Huang, W. D. , & Zhan, J. C. (2018). Influence of different sterilization treatments on the color and anthocyanin contents of mulberry juice during refrigerated storage. Innovative Food Science & Emerging Technologies, 48, 1–10. 10.1016/j.ifset.2018.05.007

[fsn32590-bib-0038] Yuan, Q. , & Zhao, L. (2017). The mulberry (*Morus* *alba L*.) fruit‐A review of characteristic components and health benefits. Journal of Agricultural and Food Chemistry, 65(48), 10383–10394. 10.1021/acs.jafc.7b03614 29129054

[fsn32590-bib-0039] Zhang, H. X. , Ma, Z. , Luo, X. Q. , & Li, X. L. (2018). Effects of mulberry fruit (*Morus* *alba L*.) consumption on health outcomes: A mini‐review. Antioxidants, 7(5), 69–82. 10.3390/antiox7050069 PMC5981255 29883416

[fsn32590-bib-0040] Zhang, H. , Yang, Y. F. , & Zhou, Z. Q. (2018). Phenolic and flavonoid contents of mandarin (*Citrus* *reticulata* *Blanco*) fruit tissues and their antioxidant capacity as evaluated by DPPH and ABTS methods. Journal of Integrative Agriculture, 17(1), 256–263. 10.1016/S2095-3119(17)61664-2

[fsn32590-bib-0041] Zhang, Y. , Butelli, E. , & Martin, C. (2014). Engineering anthocyanin biosynthesis in plants. Current Opinion in Plant Biology, 19, 81–90. 10.1016/j.pbi.2014.05.011 24907528

[fsn32590-bib-0042] Zhang, Y. , Ren, C. , Lu, G. , Cui, W. , Mu, Z. , Gao, H. , & Wang, Y. (2014). Purification, characterization and anti‐diabetic activity of a polysaccharide from mulberry leaf. Regulatory Toxicology and Pharmacology, 70(3), 687–695. 10.1016/j.yrtph.2014.10.006 25455227

[fsn32590-bib-0043] Zhou, Y. , Wang, R. , Zhang, Y. , Yang, Y. , Sun, X. , Zhang, Q. , & Yang, N. (2020). Biotransformation of phenolics and metabolites and the change in antioxidant activity in kiwifruit induced by *Lactobacillus* *plantarum* fermentation. Journal of the Science of Food and Agriculture, 100(8), 3283–3290. 10.1002/jsfa.10272 31960435

